# Supplementary material for: Spermidine as a Potential Protective Agents Against Poly(I:C)-Induced Immune Response, Oxidative Stress, Apoptosis, and Testosterone Decrease in Yak Leydig Cells
Source: Int J Mol Sci. 2025 Mar 19;26(6):2753. doi: 10.3390/ijms26062753 (PMC11942872; doi:10.3390/ijms26062753)
Supplement: Supplementary file 1 [file ijms-26-02753-s001.zip › supplememtary File S1.pdf]

Spermidine as a Potential Protective Agents Against Poly(I:C)-Induced Immune Response, Oxidative Stress, Apoptosis, and Testosterone Decrease in Yak Leydig Cells

Yujun Tang [1,†], Hao Li [1,†], Yutian Zeng [1], Cuiting Yang [1], Run Zhang [1], Arab Khan Lund [1,2] and Ming Zhang [1,3,4,*]

1. College of Animal Science and Technology, Sichuan Agricultural University, Chengdu 611130, China
2. Faculty of Animal Production and Technology, Shaheed Benazir Bhutto University of Veterinary and Animal Science, Sakrand 67210, Pakistan
3. Key Laboratory of Livestock and Poultry Multi-Omics, Ministry of Agriculture and Rural Affairs, College of Animal Science and Technology, Sichuan Agricultural University, Chengdu 611130, China
4. Farm Animal Genetic Resources Exploration and Innovation Key Laboratory of Sichuan Province, College of Animal Science and Technology, Sichuan Agricultural University, Chengdu 611130, China

* Correspondence: zhangming@sicau.edu.cn
† These authors contributed equally to this work.

Supplementary Figures and Table

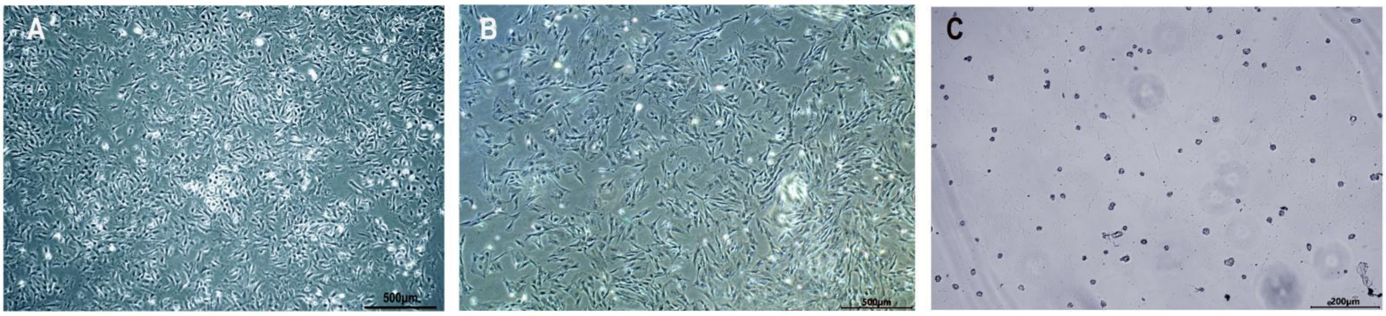

Figure S1: Culture and identification of Leydig Cells. A: Primary cell, B: Passsaging cell, C: Leydig cells with 3β-HSD staining

Table S1 Mapping Rate of transcription sequence

sample	total_reads	total_map	unique_map	positive_map	negative_map	proper_map
Con_1	47828696	44195123(92.4%)	42946436(89.79%)	21468270(44.89%)	21478166(44.91%)	39708846(83.02%)
Con_2	48885620	45122052(92.3%)	43826571(89.65%)	21915067(44.83%)	21911504(44.82%)	41233204(84.35%)
Con_3	49625410	45280852(91.25%)	43969499(88.6%)	21987857(44.31%)	21981642(44.3%)	40733248(82.08%)
Poly1	49196936	45321207(92.12%)	43973273(89.38%)	21991128(44.7%)	21982145(44.68%)	40986074(83.31%)
Poly2	46152856	42777042(92.69%)	41512562(89.95%)	20769911(45.0%)	20742651(44.94%)	38978002(84.45%)
Poly3	47221490	43727498(92.6%)	42413339(89.82%)	21225099(44.95%)	21188240(44.87%)	39579054(83.82%)
Spd1	51312296	47277241(92.14%)	45894051(89.44%)	22928173(44.68%)	22965878(44.76%)	43457906(84.69%)
Spd2	45184066	41612723(92.1%)	40353896(89.31%)	20158282(44.61%)	20195614(44.7%)	38293310(84.75%)
Spd3	47730260	44072914(92.34%)	42695395(89.45%)	21316837(44.66%)	21378558(44.79%)	40414056(84.67%)

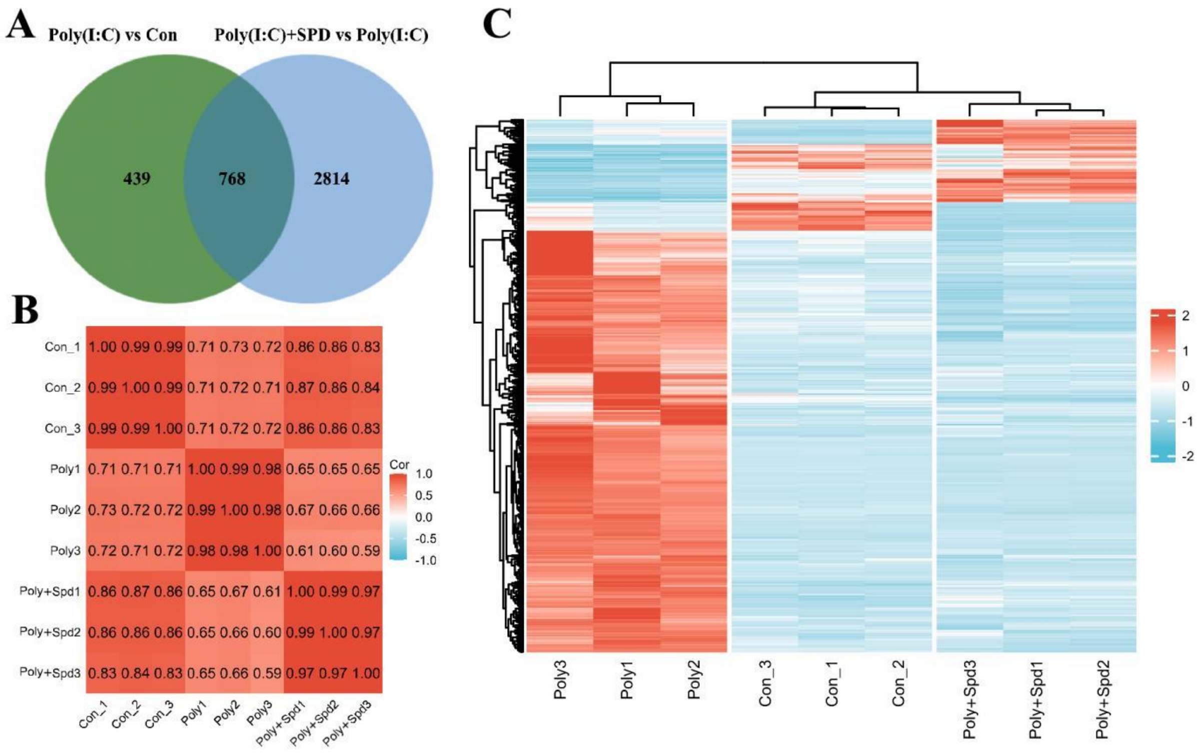

Figure S2: The data quality control from transcritome. A.The Venn diagram illustrates the overlap of differentially expressed genes (DEGs) between two comparisons: Poly(I:C) vs CON and SPDvsPoly(I:C). A total of 768 genes are co-expressed DEGs; B. the pearson's correlation of DEGs in three groups; C. the heatmap of DEGs.
